# Qualitative Evidence from Studies of Interventions Aimed at Return to Work and Staying at Work for Persons with Chronic Musculoskeletal Pain

**DOI:** 10.3390/jcm10061247

**Published:** 2021-03-17

**Authors:** Gunilla M. Liedberg, Mathilda Björk, Elena Dragioti, Christina Turesson

**Affiliations:** 1 Pain and Rehabilitation Centre, Department of Health, Medicine and Caring Sciences, Linköping University, 58185 Linköping, Sweden; gunilla.liedberg@liu.se (G.M.L.); elena.dragioti@liu.se (E.D.); 2 Department of Health, Medicine and Caring Sciences, Division of Prevention, Rehabilitation and Community Medicine, Linköping University, 58183 Linköping, Sweden; christina.turesson@liu.se

**Keywords:** chronic pain, interventions, evidence assessment, occupation, qualitative review, rehabilitation, return to work, systematic review

## Abstract

Chronic musculoskeletal pain is a significant burden for employees, employers, and society. However, more knowledge is needed about which interventions reduce sick leave. Interventions were defined as the act or an instance of intervening, provided by different stakeholders. This review synthesizes the experiences of patients, employers, and health professionals concerning the interventions that influence returning to work and staying at work for persons with chronic musculoskeletal pain. A literature search was performed using several combinations of key terms. Overall, 18 qualitative studies published between 2002 and 2018 were included. Qualitative analysis assessed how much confidence could be placed in each review finding. Moderate evidence was found for factors improving the return to work process such as collaboration between stakeholders, including the persons with chronic musculoskeletal pain and support from all involved actors in the process. Moderate evidence was found for self-management strategies and workplace adjustments needed to facilitate more persons to returning to work and staying at work despite pain. This review provides stakeholders, employers, and health professionals’ information that could be used to develop and implement interventions to increase the possibilities for persons with chronic musculoskeletal pain returning to work or staying at work.

## 1. Introduction

Chronic musculoskeletal pain (CMSP) (i.e., pain duration >3 months) such as chronic neck/shoulder and back pain or generalized widespread pain (including fibromyalgia (FM)) has a prevalence from 10.4% [1] to 20% among adults [2,3,4]. CMSP negatively impacts daily activities, including employment and number of lost work days [3]. CMSP has a substantial negative impact on work-related outcomes for employees as well as a loss of productivity for society, employers, and employees [5]. Limited interventions for return to work (RTW) affect a country’s economy due to a reduced work capacity and decreased productivity and is associated with personal suffering [6].

Health professionals’ perspectives on approaches to support people with CMSP to RTW showed that RTW processes are delayed due to the way the system (all involved actors in a RTW process) is organized—i.e., a rigid system caused by a lack of coordination and collaboration is a barrier to RTW interventions and ultimately RTW [7]. Congruence between stakeholders and patient perspectives in sharing decisions on plans and goals for RTW can lead to better treatment and ultimately better outcomes. All stakeholders need to understand their roles and responsibilities in the RTW process, and communication and coordination among stakeholders is of the greatest importance [7].

A meta-ethnographical review study performed with persons with chronic pain and by employers [8] examining 41 studies showed that at the same time as managing work relationships and making workplace adjustments, health and pain representations were major challenges that had not been highlighted in previously published reviews. Pain can negatively affect workplace relationships and result in problems associated with employees requiring adjustments to their work, which are both aspects that are vital for RTW. For example, de Vries et al. [9] found that personal adjustments, for instance the possibility to decide your own work schedule, retraining for other jobs, and high perceived support from colleagues and supervisors, as well as workplace interventions were important for persons to stay at work (SAW) despite CMSP. In addition, several workplace-based interventions have been reported as important for RTW: early contact with the worker by the workplace; an offer of work accommodations; contact between the health care provider and the workplace; work site visits to assess ergonomic conditions; supernumerary replacements; and RTW coordination [10]. These interventions were investigated regarding the effect on work disability duration, economic analyses, and quality of life outcomes. Strong evidence was found that the duration of work disability is significantly reduced by work accommodations and contact between health care providers and the workplace.

Despite existing qualitative reviews, no research has examined the experiences of persons with CMSP, employers, and health care professionals in the same review concerning the effectiveness of RTW interventions. To increase motivation and adherence, it is essential to determine which RTW interventions are regarded as effective by the employees who use them. Therefore, this study synthesizes the qualitative literature reporting the experiences of persons with CMSP, employers, and health professionals to determine which interventions are most effective in terms of RTW and SAW. In addition, this study systematically synthesizes qualitative evidence related to the findings, extending the findings from previous reviews.

## 2. Materials and Methods

The Enhancing transparency in reporting the synthesis of qualitative research (ENTREQ) guidelines were used to demonstrate the selection processes and results [11]. The ENTREQ checklist consists of 21 items grouped into five main domains: introduction, methods and methodology, literature search and selection, appraisal, and synthesis of findings, and these are presented in Appendix A.

### 2.1. Eligibility Criteria

The included studies collected qualitative data on the experiences of persons with CMSP, employers, and health care professionals regarding which interventions facilitated employee RTW and SAW. Study designs were qualitative and mixed methods. For mixed method, only the qualitative part was included. Studies were included if they fulfilled all the following inclusion criteria: persons with chronic musculoskeletal pain lasting more than three months [12]; a clinical diagnosis related to chronic musculoskeletal pain (e.g., chronic neck/shoulder and low back pain or generalized widespread pain including FM); adults of working age (16–67 years); on sick leave; participating in a rehabilitation program or in paid jobs irrespective of position or organization; and own experience with RTW or SAW interventions. No restriction was made concerning gender. In addition, studies were included that described experiences of employers with the RTW process as well as health care professionals with experience treating individuals with CMSP. In this study, RTW interventions were defined as the act or an instance of intervening, provided by different stakeholders and described by the participants as focusing on facilitating/hindering a RTW/SAW.

### 2.2. Literature Search

**Figure 1:**
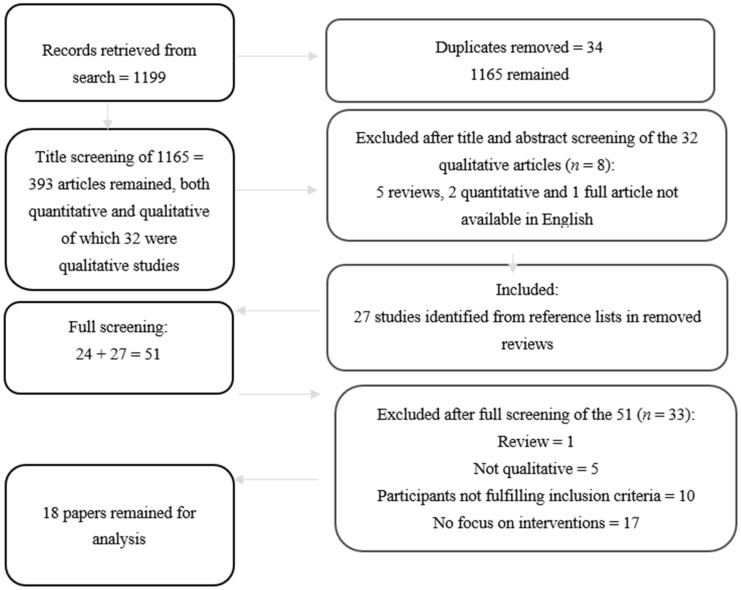
Flowchart of the search and study selection process.

A comprehensive literature search was performed in Web of Knowledge database incorporating also PubMed and with the combination of the following search key terms: return to work, chronic pain, chronic pain intervention, sick leave, vocational rehabilitation, rehabilitation, work, and work rehabilitation. The search of the databases, performed by one investigator (ED), resulted in 1199 quantitative and qualitative publications. After two independent investigators (ED and GML) screened the titles and if necessary abstracts, 393 publications remained. Of the abstracts screened, 32 were regarded as qualitative publications. Of these 32, eight were excluded: five reviews, two not including qualitative methodology and one not written in English. Altogether, 24 studies were selected for screening. Furthermore, references of relevant published systematic reviews and meta-synthesis/meta-ethnographies were hand-searched to identify publications missed by the electronic search, resulting in 27 publications added for screening. In all, 51 studies were screened and read in full (GML and MB). This screening resulted in the exclusion of 33 articles. At this stage, reasons for exclusion were noted for each paper: reviews, quantitative publications, participants not fulfilling criteria for inclusion, and no focus on interventions in the RTW/SAW process. In the end, the selection process resulted in the inclusion of 18 qualitative studies. A full report of the selection process can be seen in the flow chart diagram in Figure 1. A protocol of synthesis of the quantitative studies from the same literature search have been published elsewhere [13].

### 2.3. Quality Assessment

The quality of the 18 included studies was assessed using a qualitative research appraisal tool, the Critical Appraisal Skills Programme (CASP) [14]. The CASP checklists include ten questions covering rigor, research methods, relevance, and research integrity. Quality assessment of all included studies was conducted by two of the authors (GML and CT). If there were rating discrepancies, the issues were discussed until consensus was reached. The assessment was used to primarily discuss the quality of included studies across the ten CASP questions, since the appropriateness of excluding qualitative studies based on quality has been questioned [15]. A sensitivity analysis [16] was used to assess the possible impact of the included studies’ quality on the review’s findings—i.e., the analysis was used to test the effect of the synthesis including and excluding findings from studies of different quality.

### 2.4. Analysis

The three steps suggested by Thomas and Harden [16] for thematic synthesis were followed: coding the text, developing the descriptive themes, and producing analytical themes. The analysis started with reading and rereading the studies to form a general impression of the concepts. The Results/Findings sections of each paper were extracted and placed into Open Code software [17] and underwent line-by-line coding (step 1) where meaning units were collected and condensed into codes. This step was initially performed by one of the authors (GML) before another author (CT) repeated the process. In step 2, codes were considered and grouped according to their similarities, and descriptive second-order themes were formed. In step 3, the analytical themes were constructed to accurately analyze the themes, to go “beyond” [16] the original studies, and to form the third-order themes. CT and GML performed steps 2 and 3. All authors read, analyzed, and discussed the material until agreement was achieved.

Five third-order themes emerged (Table 1) that influenced the RTW and SAW processes in different ways: Societal structures influencing interventions; Participating professionals’ approach; The need of support; Parameters for personal change of behavior; and Facilitating interventions at the workplace. These third-order themes contained 19 s-order themes.

### 2.5. Confidence Assessment

The strength of the evidence of the emerging second-order themes from the qualitative analysis was evaluated by GML and CT using the Confidence in Evidence from Reviews of Qualitative research (CERQual) [36]. CERQual assesses how much confidence can be placed in each review finding—i.e., a second-order theme. This is an assessment of the extent to which a review finding is a reasonable representation of the phenomenon of interest, in this case, interventions of importance for RTW or SAW. The tool considers four components: (1) methodological limitations—problems in the design or conduct based on quality appraisal using in this case the CASP; (2) coherence—the extent to which findings are grounded in data from contributing primary studies; (3) adequacy of data—degree of richness and quantity of data supporting the findings; and (4) relevance—the extent to which the body of evidence from the primary studies supporting a review finding is applicable to the context, perspective, or population, phenomenon of interest, or setting. To indicate the certainty of qualitative evidence, four levels can be used: high, moderate, low, and very low confidence. Lewin et al. [36] defines the confidence levels as follows: high confidence indicates it is highly likely that the review finding is a reasonable representation of the phenomenon of interest; moderate confidence indicates that it is likely that the review finding is a reasonable representation; low level confidence indicates that it is possible that the review finding is a reasonable representation of the phenomenon of interest; and very low level confidence indicates that it is unclear whether the review finding is a reasonable representation.

## 3. Results

### 3.1. Study Characteristics

Table 2 summarizes the 18 included studies (one from South Africa, one from Asia, one from Australia, two from North America, and 13 from Europe), which were published between 2002 and 2018: 12 focused on patients; three focused on patients and health care professionals; two focused on patients and employers; and one focused on health care professionals. Three of these studies used the same sample, but the aims of these studies differed, so the participants from these studies were included in our analysis. However, the sample from these three studies was only included once in the total number of study participants. In total, 504 participants were included in the studies: 299 patients (127 males and 172 females), 187 health care professionals, and 18 employers.

### 3.2. Assessment of Quality and Level of Evidence

Quality assessments of the 18 included studies were performed: 13 studies were rated as good quality and five were rated as moderate quality. A summary from the CASP quality appraisal can be found in Table 3.

The confidence of evidence varied between the review findings from low to moderate. Of the 19 s-order review findings, 14 were rated as moderate and five were rated as low confidence (Table 4).

### 3.3. Findings

The results show that overall factors and specific interventions were described at different levels both at a societal and structural level as well as more individual measures especially in connection with rehabilitation programs. Five third-order themes emerged (Table 1) that influenced the RTW and SAW processes in different ways: Societal structures influencing interventions; Participating professionals’ approach; The need of support; Parameters for personal change of behavior; and Facilitating interventions in the workplace. These third-order themes contain 19 s-order themes, which are identified by underlining in the text below. In addition, the findings are exemplified by some of the included studies in the following section.

#### 3.3.1. Societal Structures Influencing Interventions

Cultural values in society were described in more than half of the included articles, signifying their importance for RTW and SAW [18,19,20,21,22,23,24,25,26,27]. The value society attaches to the concept of work often encourages people to remain in the labor market, as staying outside the labor market may imply that someone is outside society. Being doubted or judged by stakeholders, including employers, was described [18,22,27] as a negative experience for people with chronic pain and defined as a lack of support from society. Other values in society connected to work and regarded as important for the worker were social contacts, a feeling of responsibility, loyalty toward colleagues, self-respect, and a way to accomplish self-realization [20,21,24,25,26,27].

Work restructuring because of ongoing societal changes in society, for example, in the form of a changed economic climate in the labor market also influenced RTW and SAW. Increased expectations of their ability to work overtime and to work at a faster pace and a fear that work tasks will increase and become more difficult caused stress and became barriers to RTW and SAW [20,25]. The form of employment as a facilitating/hindering factor was described. For example, being self-employed sometimes was regarded as an advantage, although the negative effects associated with self-employment such as the reduced possibility of adjustments were also emphasized [28,29]. Environmental barriers in the form of legal aspects such as the need for the employers to be aware and have knowledge of their obligations and increased costs needed to support employees and employers [30] may be a consequence of inadequate workplace policy [27], weak employment law, and government recommendations [30].

#### 3.3.2. Participating Professionals’ Approach

In more than half the articles, the way in which agency, social welfare, employers, and health care professionals exercise their role implementing interventions was described as a major factor for determining the success of RTW.

In several studies [18,20,21,22,23,24,27,31,32,33], the relationships between the employer and the employee were seen as a facilitator or a barrier in RTW. Employers stated that it was helpful to trust employees, and maintaining contact with absent employees was valuable [22,23,33]. The participating employers in Jacobsen and Lillefjell’s study [23] clearly identified a need for extended and closer collaboration between employees, employers, and rehabilitation professionals.

Good inter-personal collaboration between the stakeholders (i.e., all professionals included in the RTW process) was seen as positive [18,22,23,24,27,30,31,32,33] and confirmed that the rehabilitation process was regularly supervised and therefore fostered a quicker RTW [18,22,23,24,27,32]. More contact with professionals was also seen as a way to successfully influence the RTW process [19,22,24,27,28,31]. In addition, positive encounters (e.g., during meetings) that considered a person’s situation seriously, reflecting trust in the person’s judgment and respect for their views regarding their pain, were also considered facilitators for RTW and SAW [18,21,22,32,33]. Additionally, transfer of information/knowledge between health professionals and stakeholders in general was emphasized [18,20,23,27,32]. For example, an external exchange of documentation as well as the agreement in reaching goals towards RTW were seen as facilitators for successful RTW [27,31]. The importance of clear, regular communication between all involved parties in the process was stated [22,23,27,32], including making assumptions explicit [33]. Patients in Soeker et al.’s study [27] reported that poor communication between professionals from different organizations resulted in conflicts between the medical professionals, employers, and the employees. The importance of communication between stakeholders were highlighted also in Coole et al.’s study [30] where both employer- and patient-dependent factors influenced the communication and therefore the process.

Finally, attitudes from stakeholders were highlighted in some of the reviewed studies as influencing the RTW process [18,19,22,24,27,31,33]. Soeker et al. [27] describe a disrespectful attitude of the medical profession, whereas Buijs et al. [31] and Hubertsson et al. [22] describe health care professionals as being understanding and treating patients with respect. On the other hand, some participants described representatives of the Swedish Social Insurance Agency in Hubertson et al.’s study [22] as providing vague or incorrect information as well as treating them disrespectfully, creating a feeling of lost human value. Being met with an unsympathetic attitude, a lack of empathy from employers, and a feeling of being doubted concerning personal abilities were seen as barriers to sustainable and successful RTW [22]. Wainright et al. [33] found that symbolic gestures of trust and value from employers in terms of fitness rather than sickness improved how the participants viewed their capacity. An uncaring attitude from the employer meant an uncaring climate at the workplace and a decrease in work productivity [18,33].

#### 3.3.3. The Need of Support

Most of the studies found that support from the surrounding environment—i.e., professionals from the different organizations involved in the RTW process, colleagues at the rehab clinic, workplace settings, family, and friends—encouraged RTW and SAW [20,21,22,23,24,25,27,28,31,32,33,34,35].

The health care system, especially primary care, was reported [22,24] as having limited assistance, and waiting times resulted in a feeling of putting life on hold [21,31]. Hubertsson et al. [22] found that the informants had a strong and explicit desire for guidance and emphasized the need for accessibility of health care providers, including psychological support and support with practical issues concerning their rehabilitation process in general. In a study by Coole et al. [28], the role of general practitioners (GP) was emphasized, as many participants reported that they had not received any helpful advice or support in relation to work—e.g., some were told to avoid work altogether. In addition, there was a lack of dialogue between GPs and employers. Other studies reported that the GPs mainly prescribed medication, and the participants questioned the value of medication, since the medications often negatively impact their ability to work [24,31].

Support from the employer was reported as a facilitator of RTW, while absence of support was considered a barrier [20,21,23,24,25,29,31]. An Australian study [32] developing self-management modules to improve vocational outcomes showed that the module ‘Managing a return to work’ included positive working relationships such as support from supervisors and colleagues [20,21,24,25,29,31,32,33,34,35]. According to Buijs et al. [31], supervisors’ lack of participation in workplace interventions and workplace adjustments were external barriers for RTW. Former participants in a rehabilitation program [21] emphasized the importance of support from colleagues at the rehabilitation clinic to facilitate self-reflection and understanding through the sharing. In addition, participants identified the importance of colleagues helping them with their work tasks when they RTW [20,25,29,31,34]. Shaw and Huang [34] found that RTW and SAW can be facilitated by avoiding activities that might cause discomfort, explaining physical limitations to colleagues and supervisors, and receiving emotional support from co-workers. Durand et al. [35] found that a work and organization culture that values support, both formal and informal from co-workers, is an indicator of the importance of the adaptation of work activity. However, some studies noted some negative effects of adjustments at the workplace, as these adaptations could raise doubts as to how long their colleagues’ support might continue and a feeling of not fulfilling their part as a member of a team [23,25,29].

The studies noted the significance of support from partner, family, and friends [20,21,22,23,25,27,32,34,35] and how this support strongly influences the RTW rate [25,27,32] as well as becoming a necessity in surviving the RTW process [22]. When identifying indicators important for a gradual RTW with participants in a work rehabilitation program and clinics [35], the life situation (e.g., access to close friends and family) of the workers appeared important. Many of the researchers expressed a balanced life situation as a prerequisite for RTW [20,21,22,23,25,32]. For some of the participating women, the ability to remain at work depends on how the family situation can be dealt with and how a balance between their experienced pain and home life and work commitments can be coordinated as well as the introduction of a changed role in family life [22,32]. In addition, deVries et al. [20], Soeker et al. [27], and Wainwright et al. [33] highlight the importance of other family members taking over chores in the home. The need for adjusting daily routines to work and the need for education on how to cope with daily activities due to injury were perceived as vital for quality of life during sick leave and may be seen as foundational for RTW [22].

#### 3.3.4. Parameters for Personal Change of Behavior

Personal parameters were mainly described in the studies where the participants underwent or had completed a rehabilitation program [18,19,20,21,23,24,31,32]. According to Durand et al. [35], personal parameters include different groups of indicators. For example, one’s own “thoughts, beliefs and attitude”, also described by Kalsi et al. [24], were identified as important for self-efficacy and RTW. Haugli et al. [21] found that an increased self-awareness facilitates a change in how people viewed their health situation. The possibility to reconstruct their own thoughts and identity was achieved during the rehabilitation program, and changing own behavior and thinking was regarded as most important for interventions and therefore RTW. Jacobsen and Lillefjell [23] highlighted the importance of the individual responsibility to make decisions and efforts to bring about changes in one’s life in accordance with Durand et al.’s [35] second indicator: physical, cognitive, and level of self-adjustment capacity. In Johnstone et al.’s [32] study, it became clear that what people need to obtain work or to keep working are personal skills, abilities, and resources—i.e., the belief (self-efficacy) that they can do their job in spite of their health and a physical capacity to perform the required duties. These findings are confirmed by Shaw and Huang [34]. Furthermore, accepting and coping with the pain and keeping a positive attitude [24,26] help build competence and reconceptualize one’s role as an employee [18]. To be compliant (i.e., the extent an employee accepts functional restoration and RTW rather than pain reduction) is fundamental in Buijs et al.’s study [31], where graded activity of unconscious motor skills or techniques were readjusted to prevent wrong postures at work. Coole et al. [30], on the other hand, stress empowerment and self-management as important facilitators of RTW.

Internal barriers to RTW were described in some studies [18,19,20,21,22,31,35]. Ahamed et al. [18] describe uncertainty about one’s ability to accomplish work tasks, feelings of inadequacy, and other people’s judgment as barriers to SAW and RTW. Furthermore, some participants expressed a fear that a recurring injury might increase their pain and restrict their ability to make a living, further impacting their psycho-social conditions [18,22]. Coole et al. [19] describe participants being unsure of what was wrong with their body, and this uncertainty led to anxiety about their future work capacity and that their condition may be regarded as progressive deterioration. Despair was found to be an important internal barrier [31], since patients with despair may externalize their problems as unsolvable and pain oriented.

#### 3.3.5. Facilitating Interventions at the Work Place

Several studies identified adjustments and strategies that accommodate a person’s physical needs as facilitators for RTW and SAW—e.g., reduction of working hours, involvement in decision making, extra time to complete tasks and schemes, regular breaks, and the possibility to change one’s posture and position [19,20,22,23,24,25,26,27,29,30,31,32,33,34,35]. In addition, several studies identified facilitators for RTW and SAW related to education in how to perform duties safely, an adapted work technique/equipment/technical aids/assistive device, the possibility to work as supernumerary, gradually being reintroduced into the workplace, trying alternating duties/tasks, and retraining in other jobs [18,19,20,25,26,31,32,34,35]. For some employees, a reduction of working hours may imply economic consequences, but for others, this may mean that they can no longer maintain the same responsibility and work tasks as before [29]. In some cases, the unwillingness of employers to modify employees’ workload led to an informal basis of adjustments, either by themselves or by involving colleagues [20,23].

## 4. Discussion

This review primarily focuses on the synthesis of the qualitative literature reporting the experiences of people with CMSP, employers, and health professionals about interventions aimed at RTW and SAW. Overall, the interventions in our study emphasized how the social and structural levels influence the RTW and SAW process. This influence was summarized into five third-order themes: Societal structures influencing interventions; Participant professionals’ impact; The need of support; Parameters for personal change of behavior; and Facilitating interventions at the workplace.

The findings are based on the fact that 14 of the 19 s-order themes in our results were assessed as having a moderate confidence—i.e., the findings are likely to be a reasonable representation of the phenomenon of interest. The remaining five second-order themes were assessed as having low confidence, although still carrying significant value. Despite the relatively large number of studies, we followed the directives [36] for the assessment of evidence very strictly. Moreover, as all the studies had some form of limitation either in method, adequacy, or relevance, no review finding was assessed as having high confidence.

Several studies identified and synthesized in this this study reported that cultural values, one of the second-order themes, influenced the RTW interventions. Similarly, Grant [8] concluded that one limitation of RTW interventions is that societal expectations about work were regarded as both facilitators and barriers, but the estimated confidence of this evidence was low, whereas our study found the estimated evidence that cultural values were both facilitators and barriers to be moderate. Toye [37] describes the need for cultural transformation in the way people and health professionals view pain to no longer focus on the pain experience itself but to focus on active behaviors/interventions that can lead to a RTW/SAW. This is to ensure that people with chronic pain can live as comfortable a life as possible. Our review, in line with underlying values described by Seing et al. [38], found that meetings regarding work ability and RTW with stakeholders who have different regulations and practices revealed three perspectives: a medical perspective, a workplace perspective, and a regulatory perspective. The authors conclude that collaboration can be difficult to implement between organizations because of different regulations, goals, and guidelines. This conclusion is also found in a novel study by Svanholm et al. [39], which highlights the need for an improved and tailored collaboration with the patient as the main actor.

As with our findings, Müssener et al. [40] found that an encouraging and supportive attitude from professionals was important for empowering persons to manage obstacles during the rehabilitation process. In our study, the lack of collaboration between the persons with CMSP and different stakeholders and among stakeholders negatively influenced the process. Furthermore, communication and transfer of information/knowledge between stakeholders were emphasized as improving the process and would be valuable for creating standards of practice to improve the process of RTW, which are findings also confirmed by Magalhães [7]. Collaboration between different actors must consider complex relationships and social hierarchies when trying to improve these inter-professional relationships. In our study, the importance of having regular contact between workplace and the worker was stated as valuable by both workers and employers participating in the studies, although the evidence was deemed to have low confidence. According to Toye [37], there is strong evidence that an employer’s role is fundamental to a successful and timely RTW and can make the process much faster. Furthermore, Seing [38] describes an unequal distribution of power between stakeholders in an observational study of stakeholders’ meetings meant to support RTW. The employers had a decisive importance as they were able to say they can or cannot adjust the work environment for the individual. Franche et al. [10] reported strong evidence that contact between health care providers and the workplace significantly reduces the duration of work disability. Furthermore, Magalhães [7] emphasized that an excessive bureaucracy in the different organizations complicate RTW, and a successful RTW requires a dynamic interdisciplinary team.

Our review generated findings from evidence with moderate confidence revealing significant importance of support from, for example, supervisors and colleagues for the possibility to RTW or SAW. Our study assessed that the evidence for health care professionals providing limited support resulting in delays in RTW and SAW had low confidence. Similarly, Toye [37] and Grant [8] describe delays and support from general practitioners related to prescribing sick notes and ignoring developing strategies for RTW. The importance of support for the RTW and SAW process from an individual’s partner, family, and friends was well described in studies included in this review and assessed to have moderate confidence. Similarly, Snippen et al. [41], investigating cognitions and behaviors of significant others (SOs) and work participation of individuals with a chronic disease found that a positive and encouraging attitude and encouraging and motivating behavior from SOs were facilitators for work participation. In our study, it was also obvious that the practical support was of major importance and facilitated a balanced life. Snippen et al. [41] assessed evidence for practical support to have low confidence, whereas our study found evidence for practical support to have moderate confidence, including the possibility to be relieved of chores. Furthermore, evidence for a balanced life situation was assessed to have moderate confidence in the present study, and interestingly, this theme consisted of 70% female respondents. One explanation may be that women in general still have the major responsibility according to society’s expectations for household work and family, children, and social relations despite having paid work. This may mean that more women express issues regarding their life situation and their sometimes-impossible solutions to establish a good life when experiencing pain [42].

The opportunity to change the behavior and thinking of persons with CSMP and improve their self-confidence and self-management lies to a high extent with the individual themselves, but it can obviously be facilitated through a rehabilitation program, as shown in this study. In our review, this was shown to have moderate confidence. However, it is important that support for continued development and retention of behavior also continues afterwards. The need for support was confirmed by Devan et al. [43] in investigating how to incorporate self-management strategies for persons with chronic pain after completion of a self-management intervention. They found out that a feeling of being empowered by incorporating self-management strategies into their daily life and support from clinicians, family, and friends was of outmost importance. Conflict with clinicians was experienced as a major obstacle to engage in the self-management process. The persons used active strategies in dealing with their day such as pacing, relaxation, cognitive behavioral strategies, counseling, and ergonomic advice. Providing a continuous support from participant professionals in the RTW process is of major concern, since the sustained efforts of self-managing can be exhausting and troublesome to maintain.

The importance of individual adaptation in the workplace for RTW and SAW was emphasized in almost all studies included in our review, and the evidence was assessed to have moderate confidence. These findings are parallel to the systematic reviews [8,10,37] that showed moderate/high confidence for evidence that suggests that ergonomic work site visits, significantly reduced work disability duration, and adaptations of jobs or work conditions facilitated the RTW process. Probably, the interventions most easily put into action depend on the employer, colleagues, and foremost the worker in developing a positive climate at the workplace.

### Methodological Strengths and Limitations

Our review used well-established systematic review methods concerning search, screening, and analysis for the assessment of methodological quality and evidence. Although every effort was made to identify relevant studies and a systematic search as well as a hand-search for studies were carried out, it is possible that some studies were missed.

To minimize the bias during data extraction and syntheses, we established various strategies, such as the iterative process of coding, discussions between the researchers involved, and structured discussions in case of disagreements. Researchers involved in this study had different professional backgrounds, which enriched our data synthesis. We presented our results both in a general text and in tables. This detailed reporting of the categories and the comparison with the other included studies increases the readers’ possibilities to access the reliability, according to Korhonen et al. [44], of the review and the application of its results in practice.

The RTW interventions reviewed were aimed at people on sick leave, participating in rehabilitation programs, or working, in a range of contexts and settings also present in this study globally, further strengthening the findings. Exploring patients’, employers’, and health care professionals’ accounts also provided an understanding of interventions in the RTW and SAW process from multiple perspectives with suggestions for improvement and implications. In the present review, we also included studies focusing on SAW despite CMSP. In this way, we captured aspects regarding how people manage to work despite pain, such as the possibility for personal adjustments and workplace interventions of major importance. There were very few studies found concerning SAW and CMSP, and the results from the studies show that in general, interventions that make work possible also were the interventions that were suggested for people struggling to RTW described in the other studies.

The included papers capture a span of 16 years of qualitative research from different countries exploring factors influencing the RTW process for persons with CMSP. The included studies only had minor limitations, so the findings and conclusions carry considerable weight. Common limitations in the included studies were lack of information in relation to quotations (i.e., who expressed what) and a missing discussion on the potential influence of the researchers’ positions on their results. We discussed and judged the possible impact from the included studies on the review’s findings and therefore did not exclude any studies fulfilling our inclusion criteria based on their degree of quality.

Among the participants in our included studies, there was a slight skew toward women with CMSP, but this skewness was mainly the result of two studies that included only women. The relatively high number of health professionals was the result of one questionnaire study using a mixed method design, which included 154 occupational therapists, skewing the number of health professionals. Most the studies’ participants were diagnosed with CMSP (12 studies) rather than employers or health professionals. This highlights the importance of including these groups in future research to a higher extent, as their views are of great importance for how implementation of interventions should take place.

## 5. Clinical Implications

This review provides evidence-based information based on qualitative experiences that may support stakeholders involved in the RTW process and health professionals and policy-makers in developing and improving the RTW process. In conclusion, the clinical implications are as follows.

Cultural values in society regarding work and supporting societal structures such as workplace policies or forms of employment are highly important for RTW and SAW and can promote people to return/remain in the labor market.

- Improved collaboration between stakeholders is essential, where the perspectives from the health care system, the employer, and the policy representative must be considered and intertwined when supporting an individual’s RTW process.
- It is necessary to enable active and regular support in the form of recurring meetings among the individual, the employer, and other stakeholders during the RTW process.
- There is still a need to visualize and facilitate women’s complete life situation and accompanying opportunities for returning to or staying in work, and this should be considered by all involved stakeholders in the process.
- Supporting increased self-awareness and promoting change in behavior, thinking, and level of self-adjustment capacity are key facilitators of RTW/SAW.

A tailored adaptation to the individual’s needs at the work place is crucial for the success of RTW and needs to be emphasized as a mandatory intervention and supported by health professionals/occupational health care.

## 6. Conclusions

In conclusion, our systematic review of qualitative studies produces generalizable and reliable information about the experiences of patients with CMSP, employers, and health professionals with interventions related to the RTW and SAW processes. The results can be used to support practical work and decision making for all included actors in the RTW process and therefore shorten the RTW process for persons with chronic pain. This review highlights the need for improved collaboration that includes a positive approach between involved actors and open and regular communication within the health care system, other actors in the field, and the people with CMSP. Changes at the system level might be necessary to improve the RTW process and to increase the knowledge about CMSP and its consequences.

## Figures and Tables

**Table 1:** Thematic synthesis procedure and corresponding references.

Third-Order Theme	Second-Order Theme	References
Societal structures influencing interventions	Cultural values concerning disability and a work role	[18,19,20,21,22,23,24,25,26,27]
	Ongoing societal changes/development	[20,25,28,29]
	Inadequate work place policy/guidelines	[27,30]
Participating professionals’ approach	Relationship between employer and employee	[18,20,21,22,23,24,27,31,32,33]
	Collaboration between professionals	[18,22,23,24,27,30,31,32,33]
	Contact in a higher degree with diverse professionals	[18,21,22,32,33,34]
	Encounters	[18,21,22,32,33]
	Information/knowledge	[18,20,23,27,32]
	between involved actors	
	Communication	[22,27,30,32,33]
	Attitudes	[18,19,22,24,27,31,33]
The need of support	Support from:	
	Health care	[22,24,28,31]
	Employers	[20,21,23,24,25,29,31]
	Supervisors and colleagues	[20,21,24,25,29,31,32,34]
	Partner, family, and friends	[20,21,22,23,25,27,32,34,35]
	Support is needed for a balanced life situation	[20,21,22,23,25,32,34]
Parameters for personal change of behavior	Changing own behavior and thinking	[18,21,23,24,35]
	Individual responsibility	[18,23,24,26,30,31,32,34]
	Internal barriers	[18,19,20,21,22,31,35]
Facilitating interventions at the work place	Adjustments and strategies at the workplace	[18,19,20,22,23,24,25,26,27,29,30,31,32,33,34,35]

**Table 2:** Summary of characteristics of included papers.

Author *, Country, Year Published [Reference Number]	Aim	Sample: Number, Gender, Diagnosis	Data Collection/Analysis	Context
Ahamed et al., India, 2018 [18]	To extract patient’s perspectives and understandings of barriers, facilitators, and adaptive procedures that influenced their capability to continue their empoyee-roles	10 male, 5 female. Back pain (BP)	Focus groups/ Thematically analysis	15 former patients, where 10 were employed and 5 unemployed
Buijs et al, NL, 2009 [31]	To qualitatively explore how patients and health care providers perceive the program effectiveness and which factors influence its implementation	9 male, 11 female. 12 Health care providers Low back pain (LBP)	In depth semi-structured interviews and focus groups/ Constant comparison method	Multidisciplinary outpatient care case management (MOC) program
Coole et al., UK, 2014 [30]	To explore the experiences of Occupational Therapists (OT) in communicating with the employers of patients with musculoskeletal conditions.	154 occupational therapists	Mixed method. Questionnaires with open questions/Thematically analysis	Occupational therapists working to support return to and maintenance of for instance work
Coole et al., UK, 2010 [28]	To explore the experiences of employed people with back pain regarding the help they have received from GPs and other clinicians regarding work	12 male, 13 female. LBP	Semi-structured individual interviews/Thematic analysis	Employed persons participating in Back Pain Rehabilitation (BPR)
Coole et al., UK, 2010 [29]	To explore employed patients’ experiences and perceptions of work, prior to attending a rehab program	12 male, 13 female. LBP	Semi-structured individual interviews/Thematic analysis	Employed persons participating in (BPR)
Coole et al., UK, 2010 [19]	To explore the individual experiences and perceptions of patients awaiting rehabilitation who were concerned about their ability to work because of persisting, or recurrent, low back pain	12 male, 13 female. LBP	Semi-structured individual interviews/Thematic analysis	Employed persons participating in (BPR)
de Vries et al., NL, 2012 [20]	To explore why people with CMP stay at work despite pain (motivators) and how they manage to maintain working (success factors)	9 male, 12 female. Chronic nonspecific musculoskeletal pain (CMP)	Semi-structured interviews/Thematic analysis	Persons who stayed at work despite CMP
Durand et al., Canada,2009 [35]	To identify indicators of the margin of maneuver taken into account during the gradual RTW of individuals involved in a musculoskeletal disorders (MSD)-related disability situation	9 male, 2 female. 9 clinicians/experts. CMP	Individual interviews and group interview/Content analysis	Participants in a work rehabilitation program and the clinician who was managing the worker.
Haugli et al., Norway, 2011 [21]	To explore the individual experiences regarding important elements of the rehabilitation program that might have contributed to a successful RTW 3 years after completing the program.	6 male, 14 female. Musculoskeletal disorders (MSD)	Semi-structured telephone interviews/Giorgi’s phenomenological analysis	Persons who attended an occupational rehabilitation program 3 years earlier
Hubertsson et al., Sweden, 2011 [22]	To study how patients’ with experience of sickness absence due to MSD have perceived their contact with the SIA and the health care system, and what factors can be described as facilitating or obstructing recovery and return to work.	4 male, 11 female (MSD)	In-depth individual interviews/Latent content analysis	Had to be in sick leave due to musculoskeletal disorders for a minimum of 6 months in total over the past three years
Jacobsen and Lillefjell, Norway, 2014 [23]	To identify factors important to promote a successful RTW as experienced by employers and employees with CMP who have been on sick leave	2 males, 4 females. 5 Employers. CMP	Interviews/Giorgis phenomenological analysis	Attending a 12-week rehabilitation program
Johnstone et al., Australia, 2014 [32]	To develop and evaluate the content of two self-management training modules to improve vocational outcomes for those with chronic musculoskeletal disorders (CMD)	6 males, 2 females. 12 rehab professional Chronic musculoskeletal disorders (CMD)	Focus groups/Concept-mapping sessions	Attending a Chronic Disease Self-Management Program
Kalsi et al., UK, 2016 [24]	To explore patients’ beliefs and attitudes toward return to work (RTW) and understand how these may impact on RTW readiness	8 males, 9 females Chronic pain (CP)	Focus groups/Thematic analysis	3 weeks high-intensity pain management rehabilitation program
Liedberg and Henriksson, Sweden, 2002 [25]	To examine which factors women with FM perceive as influencing their capacity to remain in a work role	39 females Fibromyalgia (FM)	Individual ínterviews/ Content analysis	Working and non-working women with FM previously participated in a questionnaire study
Löfgren et al., Sweden, 2006 [26]	How women with FM managed to work in spite of their difficulties	12 females. FM	Diaries, focus groups and individual interviews/Content analysis and grounded theory	Women with FM working or studying 6 years after their rehabilitation
Shaw and Huang, US, 2005 [34]	To identify themes related to self-efficacy and outcome expectancy for RTW	26 male, 25 female LBP	Individual interviews and focus groups/Content analysis	Participants who had returned to work and individuals receiving regular physiotherapy treatments
Soeker et al., South Africa, 2008 [27]	To elicit perceptions and experiences of facilitators and barriers that affected individuals who received back rehabilitation and their ability to resume their work role	18 males, 8 females BP	Focus groups/Thematic analysis	Patients drawn from an Occupational therapy department and/or a rehabilitation clinic
Wainwright et al., UK, 2013 [33]	To investigate employers’and employees´experiences of managing RTW when someone has taken sick leave for chronic pain and to explore the perceived efficacy of the fit note	Employees 8 males, 5 females Employers 13 CP	Semi structured individual interviews/Grounded theory	Employees had to be in employment, needed a sick/fit note last year, or be on current sick leave. Employers had to have experiences of managing sick leave for an employee with chronic pain.

* Only the first and second author are indicated by name; more than two authors are indicated by et al.

**Table 3:** Overall presentation from the assessment of methodological quality of the 18 included studies.

Clear Aim	Method Appropriate	Research Design Appropriate	Appropriate Recruitment Strategy	Data Collection	Relationship Adequately Considered	Ethical Issues Considered	Data Analysis	Clear Statements of Findings	Value of the Research
18 Yes	18 Yes	17 Yes 1 Can’t tell	18 Yes	18 Yes	1 Yes 17 Can’t tell	17 Yes 1 Can’t tell	17 Yes 1 Can’t tell	17 Yes 1 Can’t tell	13 Yes 5 Can’t tell

**Table 4:** Critical appraisal of review finding using Confidence in Evidence from Reviews of Qualitative research (CERQual) evaluation. The second-order themes were developed to explain the content.

Second-Order Theme	Studies Contributing to the Review Finding	CERQual Assessment of Confidence in the Evidence	Explanation of CERQual Assessment
Cultural values concerning disability and a work role in the RTW and SAW processes	[18,19,20,21,22,23,24,25,26,27]	Moderate *	The findings were relevant across different contexts/settings in five countries and three continents. Female participants with CMSP (65%) were overrepresented.
Ongoing societal changes and developments that influence the possibilities of a sustainable RTW and SAW	[20,25,28,29]	Low ***	The findings were only represented from three countries in Europe, which limited the quantity of the data and data richness and lowered the confidence. Female participants with CMSP (70%) were overrepresented.
Inadequate workplace policy/guidelines impacting the RTW process	[27,30]	Low ***	The findings were only represented from two countries, which limited the quantity of the data and data richness and lowered the confidence. Male participants with CMSP (69%) were overrepresented.
Relationship between employer and employee needs to be permeated by mutual respect to ensure successful RTW and SAW	[18,20,21,22,23,24,27,31,32,33]	Moderate *	The findings are relevant in seven countries on four continents. Equal representation between men and women.
Collaboration between professionals may foster quicker RTW	[18,22,23,24,27,30,31,32,33]	Moderate *	The findings are relevant in seven countries on four continents. Male participants with CMSP (54%) were overrepresented.
More contact between diverse professionals may affect the RTW process in a positive manner	[18,21,22,32,33,34]]	Low ***	The findings were only represented from four countries (three European countries), which limited the quantity of the data and data richness and lowered the confidence. Female participants with CMSP (51%) were overrepresented.
Encounters must be permeated by an understanding of the individual and a positive tone	[18,21,22,32,33]	Moderate *	The findings were relevant in five countries on three continents. Female participants with CMSP (52%) were overrepresented.
Information/knowledge between involved actors facilitates and accelerates RTW and SAW	[18,20,23,27,32]	Moderate *	The findings are relevant in five countries on four continents. Male participants with CMSP (59%) were overrepresented.
Communication between all included actors in the RTW process must be done continuously	[22,27,30,32,33]	Moderate *	The findings are relevant in four countries on three continents. Male participants with CMSP (58%) were overrepresented.
Attitudes must be supportive to promote a successful RTW and SAW	[18,19,22,24,27,31,33]]	Moderate *	The findings are relevant in four countries on three continents. Male participants with CMSP (53%) were overrepresented.
Health care should assist with guidance and support to facilitate RTW and SAW	[22,24,28,31]	Low ***	The findings were only represented from four European countries, which limited the quantity of the data and data richness and lowered the confidence. Female participants with CMSP (57%) were overrepresented.
Support from employers of major importance for RTW and SAW	[20,21,23,24,25,29,31]	Low ***	The findings were only represented from four European countries, which limited the quantity of the data and data richness and lowered the confidence. Female participants with CMSP (58%) were overrepresented.
Supervisors’ and colleagues’ support is important in managing RTW and SAW	[20,21,24,25,29,31,32,34]	Moderate *	The findings are relevant in seven countries on three continents. Female participants with CMSP (59%) were overrepresented.
Partner, family, and friends can strongly influence the RTW rate as well as the possibility to SAW	[20,21,22,23,25,27,32,34,35]	Moderate *	The findings are relevant in seven countries on four continents. Female participants with CMSP (60%) were overrepresented.
Support is needed for a balanced life situation, which might be a prerequisite for RTW and SAW	[20,21,22,23,25,32,34]	Moderate *	The findings are relevant in five countries on three continents. Female participants with CMSP (70%) were overrepresented.
Changing own behavior and thinking are important for RTW interventions to be successful	[18,21,23,24,35]	Moderate *	The findings are relevant in four countries on three continents. Male participants with CMSP (51%) were overrepresented.
Individual responsibility for efforts in RTW and SAW bring about changes in their daily life	[18,23,24,26,30,31,32,34]	Moderate **	The findings are relevant in seven countries on four continents. Female participants with CMSP (53%) were overrepresented.
Internal barriers such as a feeling of inadequacy must be dealt for RTW and SAW to be successful	[18,19,20,21,22,31,35]	Moderate *	The findings are relevant in five countries on three continents. Female participants with CMSP (54%) were overrepresented.
Adjustments and strategies at the work facilitate a RTW and SAW	[18,19,20,22,23,24,25,26,27,29,30,31,32,33,34,35]	Moderate *	The findings are relevant in nine countries on five continents. Female participants with CMSP (56%) were overrepresented.

* Moderate = This finding was graded as moderate confidence because of minor concerns regarding methodological limitations, coherence, adequacy, and relevance. All studies included participants of working age and the aims and contexts support the relevance. Most themes were richly described, and findings were relevant across different contexts/settings. ** Moderate = This finding was graded as moderate confidence because of no or very minor concerns regarding methodological limitations, coherence, and minor concerns regarding adequacy and relevance. All studies included participants of working age and the aims and contexts support the relevance. Most themes were richly described, and findings were relevant across different contexts/settings. *** Low = This finding was graded as low confidence because moderate concerns of adequacy. In addition, there were minor concerns regarding methodological limitations, coherence, and relevance. All studies included participants of working age, and the aims and contexts support the relevance.

## Appendix A

Enhancing transparency in reporting the synthesis of qualitative research: the ENTREQ statement.

**Table A1:** ENTREQ checklist.

No. Item	Guide Questions/Description	Reported on Page
1. Aim	State the research question the synthesis addresses	2
2. Synthesis methodology	Identify the synthesis methodology or theoretical framework which underpins the synthesis, and describe the rationale for choice of methodology (e.g. meta-ethnography, thematic synthesis, critical interpretive synthesis, grounded theory synthesis, realist synthesis, meta-aggregation, meta-study, framework synthesis)	4 Table 1.
3. Approach to searching	Indicate whether the search was pre-planned (comprehensive search strategies to seek all available studies) or iterative (to seek all available concepts until they theoretical saturation is achieved)	3
4. Inclusion criteria	Specify the inclusion/exclusion criteria (e.g., in terms of population, language, year limits, type of publication, study type)	2
5. Data sources	Describe the information sources used (e.g. electronic databases (MEDLINE, EMBASE, CINAHL, psycINFO), grey literature databases (digital thesis, policy reports), relevant organisational websites, experts, information specialists, generic web searches (Google Scholar) hand searching, reference lists) and when the searches conducted; provide the rationale for using the data source	3
6. Electronic Search strategy	Describe the literature search (e.g. provide electronic search strategies with population terms, clinical or health topic terms, experiential or social phenomena related terms, filters for qualitative research, and search limits)	3
7. Study screening methods	Describe the process of study screening and sifting (e.g. title, abstract and full text review, number of independent reviewers who screened studies)	3
8. Study characteristics	Present the characteristics of the included studies (e.g. year of publication, country, population, number of participants, data collection, methodology, analysis, research questions)	Table 2.
9. Study selection results	Identify the number of studies screened and provide reasons for study exclusion (e.g., for comprehensive searching, provide numbers of studies screened and reasons for exclusion indicated in a figure/flowchart; for iterative searching describe reasons for study exclusion and inclusion based on modifications to the research question and/or contribution to theory development)	Figure 1 Flow chart
10. Rationale for appraisal	Describe the rationale and approach used to appraise the included studies or selected findings (e.g. assessment of conduct (validity and robustness), assessment of reporting (transparency), assessment of content and utility of the findings)	4–5
11. Appraisal items	State the tools, frameworks and criteria used to appraise the studies or selected findings (e.g. Existing tools: CASP, QARI, COREQ, Mays and Pope [25]; reviewer developed tools; describe the domains assessed: research team, study design, data analysis and interpretations, reporting)	4–5
12. Appraisal process	Indicate whether the appraisal was conducted independently by more than one reviewer and if consensus was required	4
13. Appraisal results	Present results of the quality assessment and indicate which articles, if any, were weighted/excluded based on the assessment and give the rationale	Table 3 and Table 4.
14. Data extraction	Indicate which sections of the primary studies were analysed and how were the data extracted from the primary studies? (e.g. all text under the headings 4 “results /conclusions” were extracted electronically and entered into a computer software)	4
15. Software	State the computer software used, if any	4
16.Number of reviewers	Identify who was involved in coding and analysis	4
17. Coding	Describe the process for coding of data (e.g. line by line coding to search for concepts)	4 Table 1.
18. Study comparison	Describe how were comparisons made within and across studies (e.g. subsequent studies were coded into pre-existing concepts, and new concepts were created when deemed necessary)	4
19. Derivation of themes	Explain whether the process of deriving the themes or constructs was inductive or deductive	4
20. Quotations	Provide quotations from the primary studies to illustrate themes/constructs, and identify whether the quotations were participant quotations of the author’s interpretation	NA
21. Synthesis output	Present rich, compelling and useful results that go beyond a summary of the primary studies (e.g. new interpretation, models of evidence, conceptual models, analytical framework, development of a new theory or construct)	12–15 Table 4.
